# Do you really get what you are looking for? Exploring the medical call writing trend in tertiary care hospitals

**DOI:** 10.12669/pjms.36.4.1642

**Published:** 2020

**Authors:** Abdur Rahim Khan, Usman Mahboob, Najma Baseer

**Affiliations:** 1 Dr. Abdur Rahim Khan MBBS, MCPS, MD, MHPE. Associate Professor of Dermatology, Khyber Girls Medical College, Hayatabad Medical Complex Peshawar, Pakistan; 2 Dr. Usman Mahboob, MBBS, MPH, FHEA, DHPE, Fellow FAIMER. Assistant Professor in Medical Education, Institute of Health Professions Education & Research (IHPER), Khyber Medical University (KMU), Peshawar, Pakistan; 3 Dr. Najma Baseer, MBBS, PhD, MHPE. Assistant Professor in Anatomy, Institute of Basic Medical Sciences (IBMS), Khyber Medical University (KMU), Peshawar, Pakistan

**Keywords:** Doctors, Exploration, Medical call writing, Tertiary care hospitals

## Abstract

**Objective:**

To explore the trend of medical call writing by doctors working in tertiary care hospitals.

**Methods:**

A quantitative descriptive cross-sectional study was carried out to evaluate the quality of medical calls written by the doctors at three tertiary care hospitals of Peshawar, Khyber Pakhtunkhwa between June 2016 to June 2017. An instrument was developed following AMEE Guide 87. Its content validity and reliability were established by 33 consultants from twenty specialties. A total of 198 medical calls (66 each) were collected from medicine, surgery and allied specialties and evaluated on the basis of validated instrument.

**Results:**

During instrument development, six items with content Validity Ratio of 0.78 & Kappa value of 0.70 were deemed most significant in every medical call written. Among all the calls, the great majority (96% and 84.34%) mentioned the reason for referral (item 1) and history of presenting problem (item 2), respectively, while item 6 (explicit mention of the doctor who will receive the call) was addressed the least (17.6%). Item 3 (Result of physical examination) and 4 (what tests have been done/arranged by the referring doctor and a summary of the main findings) were stated in < 30% of the calls whereas item 5 (diagnosis/provisional diagnosis) was specified in less than half of the calls.

**Conclusion:**

In this study, the written medical calls of different specialties were evaluated using specifically designed six items instrument. Unfortunately, the content of medical calls assessed was found to be inadequate probably because medical call writing is not explicitly taught at under and postgraduate levels.

## INTRODUCTION

Medical call writing is an important and essential written communication skill that is routinely practiced by the doctors.^1^ It is still the most prevalent and efficient form of communication in the healthcare sector. Doctors need to have good verbal, written and interpersonal communication skills. They not only need to communicate verbally and through writing with their patients 2 but they also have to interact formally with other clinicians or diagnostic service providers for patients’ management.^3^,^4^ Written communication can be between general practitioners and consultants from various specialties working in tertiary care hospitals.^5^ The purpose of this written communication is to provide optimal care to patients. According to literature the basic principle of the referral or medical call writing is to allow the doctors of different experience, belonging to different specialties communicate with each other in order to find a solution for their patient’s problem and provide the best possible care at a precise time and place.^6^ This interaction is critical as poor communication may lead to negative outcomes such as patient dissatisfaction, compromise on patient care and unnecessary workload.^1^ Medical professionals often exhibit good verbal communication skills but their writing skills in the form of history, medical call writing, referral and consultation letters are often not as developed as their verbal communication.^7^ This incongruence may be attributed to a lack of formal training in written communication skills, which leaves these doctors to either learn by emulating their senior doctors or through trial and error.^5^-^7^

Seeking a consultant’s advice is a complex act, about which relatively little is known.^1^,^2^,^8^ Despite the fact that medical call writing is an everyday medical event,^8^ medical referral is neither considered as a part of the formal medical curriculum nor does it appear to be taught as a constituent of continuing medical education. Moreover, little consideration is given on the minimum essential information that must be mentioned in any medical call request. Therefore, we wanted to determine the essential prerequisites of a good medical call on the recommendations of consultants from various specialties. Furthermore, we wanted to explore the extent to which this information is followed in the call writing of tertiary care teaching hospitals.

## METHODS

The study was done between June 2016 to June 2017. Ethical approval (DIR/KMU-EB/EM/000184, Dated: 06-06-2016) from Khyber Medical University, Peshawar and ethical review committees of Hospital A, Hospital B and Hospital C were obtained. This study was done in two phases. In phase one, an instrument containing minimum essential information required in any medical call writing was developed following the seven steps of instrument development.^9^ The Step 1 involved literature search to determine the existing literature, which had defined and addressed the construct under consideration. As a result, a 27-items medical call writing checklist for cancer patients was found.^2^ In the second step, this 27-items checklist was given to three doctors in Dermatology Unit of ‘Hospital A’ with at-least three years clinical experience. These doctors were interviewed to elaborate on the checklist. During Step 3 & 4, the checklist was amended on the basis of findings of the literature and interviews done. Some of the items were omitted as they were pertinent only to oncology call writing. Step 5 involved validation of the instrument. The same three doctors, who were interviewed during step 2, were asked to comment on the face validity or understandability of the instrument. In sixth step the amended instrument was sent to consultants of all three teaching hospitals using convenience sampling technique. They were asked to rank each item on the basis of importance, from 0 to 4, with 4 being extremely important to 0 being not important. Based on their grading, content validity index (CVI) of each item was calculated. In addition, the Kappa statistics were also calculated for inter-rater reliability. During the final step, the instrument was pilot tested on five written medical calls that were sent to the dermatology Unit of the hospital A.

In the second phase, a quantitative descriptive study was carried out in three wards of three tertiary care teaching hospitals of Peshawar. These wards included: Dermatology Unit Hospital A, Medical Unit Hospital B, and Surgical Unit Hospital C. Purposive sampling technique was used, and sample size was calculated using Open-epi sample calculator. All medical calls written for the purpose of either making diagnosis, performing any relevant investigations, prescribing treatment or making necessary adjustments in treatment were included in the study. Those patients who were diagnosed but their medical calls were sent for the purpose of performing procedures were excluded from the study. The average number of written medical calls received by each of unit was around 2-3 calls per day, suggesting that each ward gets approximately 60 calls per month. Hence, over the period of three months, we expected these three specialties units to receive 180 calls thus making a total of 540 calls. With 95% confidence level, anticipated population proportion of 0.80, 0.05 absolute precision, and relative precision of 0.0625, the calculated sample size was 198. Therefore, the content of 198 medical calls was evaluated by comparing it with the validated checklist for essential minimum information. Data was analyzed using SPSS version 20.

## RESULTS

During the instrument development, a 23-items checklist was formulated from the available literature and after the interviews with three experienced clinicians. This checklist was then sent to 39 consultants, out of which 33 from twenty specialties of the three tertiary care hospitals responded. These consultants agreed on six-items, having CVI of 0.78 & Kappa value of 0.70 that were fundamental to every medical call (Table-I). Later, 198 medical calls, 66 from each of the three tertiary care teaching hospitals were evaluated. It was observed that among all the calls, only few contained the minimum essential information, whereas most of the calls did not mention all the items (Table-II). Item number 1- Reason for referral (95.95%) and 2 - History of presenting problem (84.34%) were mentioned the most while the remaining four items were addressed in less than 50% of the calls. Item number 6 (Explicit mention of the person who will receive the call, was stated the least (17.67%) (Table-II).

**Table-I:** Process of questionnaire development and finalization of items.

S. No	Items after initial review of questionnaire (Instrument development Step 2,3,4,5,6)	CVR (For content validity)	Multi Rater Kappa (For degree of agreement)	Finalized items having kappa value ≥ 0.70 (Instrument development Step 7)
1	Patient’s social history e.g. employment, home situation	-0.03	0.37	
2	Reason for referral	1	1.00	Reason for referral
3	History of presenting problem	0.93	0.93	History of presenting problem
4	Family history of medical problem	-0.15	0.46	
5	Past medical history – unrelated to the presenting problem	0.51	0.62	
6	Inter current medical conditions – physical & psychiatric	0.63	0.69	
7	Current medication	0.69	0.73	
8	Clinical findings: results of physical examination	0.81	0.82	Clinical findings: results of physical examination
9	What tests have been done or arranged by the referring doctor & a summary of the main findings	0.87	0.88	What tests have been done or arranged by the referring doctor & a summary of the main findings
10	Diagnosis/provisional diagnosis	0.81	0.82	Diagnosis/provisional diagnosis
11	Referring doctor’s thoughts on what may be appropriate management	0.33	0.52	
12	What other opinions have been expressed by other doctors about patient management	0.33	0.50	
13	Any factors possibly mitigating against certain treatments or treatment arrangements – medical, psycho-social, or demographic	-0.03	0.45	
14	Involvement of other doctors in the case	0.45	0.59	
15	What the patient has been told regarding diagnosis, prognosis, treatment options	0.75	0.78	
16	The patient’s wishes, expectations or concerns regarding information disclosure, decision making, treatment	-0.09	0.42	
17	Impact of the cancer & its treatment on the patient’s work, leisure and self	-0.03	0.42	
18	Any concerns about patient compliance willingness to accept advice	0.33	0.47	
19	Explicit mention of the doctor who will receive the call (Visiting Physician/ Visiting Surgeon/ Senior Registrar/ Medical Officer)	0.81	0.82	Explicit mention of the doctor who will receive the call (Visiting Physician/ Visiting Surgeon/ Senior Registrar/ Medical Officer)
20	Explicit mention of the doctor who has written the Call.	0.57	0.65	
21	Whether an interpreter is required for the consultation [if the patient has difficulty speaking some language, Pushto/Urdu etc.	0.33	0.52	
22	Information regarding any formal clinical trials the patient is on, has been offered, or is eligible for	0.33	0.50	
23	Any wishes/concerns of the patient’s family, e.g. about the disclosure of information to the patient	0.21	0.48	

When data from each of the three tertiary care hospitals was explored further, it was observed that doctors of hospital C and A addressed item 1 (reason for referral) more than the calls written in hospital B (Table-II). Item number 2 (history of presenting problem) was addressed by more than 80% of the doctors working in three tertiary care Hospitals. Only 7.5% of the medical calls of hospital A included item number 3 (Clinical findings: result of physical examination). Collectively, item 3 was mentioned in only 28% of the calls from these hospitals (Table-II). Similarly, item 4 was mentioned the least in hospital A calls while almost half (45.45%) of hospital B calls contained item number 4. Almost twice as many calls from hospital B contained item number 5 (Diagnosis/provisional diagnosis) as compared to hospital C. Although, hospital A mentioned Item number 6 (Explicit mention of the doctor who will receive the call) twice as much as compared to the remaining two hospitals; but overall, this item was addressed the least amongst all the other items (Table-II).

**Table-II:** Number of items addressed in medical calls.

Hospital	Item1 (Reason for referral)	Item 2 (History of presenting problem)	Item 3 (Clinical & physical examination findings)	Item 4 (Findings of Investigations)	Item 5 (Diagnosis/ Provisional diagnosis)	Item 6 (Mention the receiving doctor designation)

	n (%)	n (%)	n (%)	n (%)	n (%)	n (%)
Hospital A	64 (96.96)	55 (83.33)	05 (7.57)	06 (9.09)	28 (42.42)	19 (28.78)
Hospital B	61 (92.42)	53 (80.30)	20 (30.30)	30 (45.45)	41 (62.12)	08 (12.12)
Hospital C	65 (98.48)	59 (89.39)	31 (46.96)	16 (24.24)	22 (33.33)	08 (12.12)

Total	190(95.95)	167(84.34)	56(28.28)	52(26.26)	91(45.95)	35(17.67)

## DISCUSSION

This study developed a consensus on minimum information required in a written medical call and explored the trend of medical call writing in tertiary hospital activities. Doctors working in tertiary care hospitals, periphery or private clinics, practice medical call writing to encounter complex patient related issues. A well-structured medical call is beneficial not only for the patient but also for the doctors and hospital administration. It helps in better management of patients and saves doctor’s time 4 as well written referrals are often met with better replies.^5^ Moreover, patients are also saved from unnecessary duplication of investigations. Their problems are addressed in a timely manner, which further reduces their hospital stay. In addition, under and overuse of hospital resources is also avoided.^3^,^6^

The results of this study showed that item number 1-reason for referral, was mentioned in a high percentage of medical calls except a few. When these few calls were further evaluated, it was observed that their content lacked four or more than four out of six items. This suggests that the doctors writing such insufficient calls were unaware of the importance of good medical call. High percentage of reasons for referral in a medical call has been reported previously from western literature.^10^ However, a local study previously done showed that only half of the medical calls had mentioned reason for referral.^11^ Doctors need to prioritize the patients’ ‘reason for referral’ more than any other issue as those referrals which commence with a specific request are considered of high quality.^12^

In our study, history of presenting problem was mentioned in more than 80% of the calls, which is in accordance with literature.^11^,^13^ This high incidence may be attributed to the fact that patient’s history of presenting complaints is the first detail which is documented on patient’s arrival.^10^ It is further suggested that the relevant information needs to be highlighted in the medical calls to ensure better coordination via good referral care.^12^ Only one-third (28.28%) of the medical calls stated Clinical findings: results of physical examination (Item 3). This low frequency may be due to either heavy workload in these departments that prevented doctors from mentioning these detail or a lack of any structured template for medical calls.^7^,^14^ Interestingly, this item has also been reported in a relatively low percentages (17% and 36%) in previous studies.^11^,^14^

Item 4 was stated in one fourth (26.26%) of the medical calls, which was slightly better than the figures mentioned in literature (15%).^14^ The issues mentioned earlier in discussion might have also contributed to this low percentage. It is a norm in the hospital to consult on call units, which at the same instance are also running the outdoor patient departments (OPDs). Due to huge patients’ influx in these OPDs, the consulting doctors are heavily occupied. Therefore, there is a possibility of overlooking the investigations, if they are not mentioned explicitly in a call. This may lead to either duplication of investigations or a missed diagnosis. The inadequate information to a consulting doctor thus hampers or delays appropriate management of a patient.^1^,^6^,^13^

The item 5 (diagnosis and provisional diagnosis) was mentioned in approximately half of the calls, which was similar to the findings of Sardella *et al*.^14^ Either the reporting doctors prefer to refrain from making an erroneous diagnosis or are unable to link their diagnosis with the underlying issue for which the medical call is being written.^14^ Interestingly the item 6, which requires an explicit mention of the doctor being addressed in the call, was stated the least. Lack of this information may bear serious consequences on patient’s health.^4^,^12^ Therefore, due to lack of training in medical call writing, the referring doctors are usually unaware of the significance of written communication skills in multidisciplinary care.^1^

### Limitations of the study

We could not evaluate medical calls written by general practitioners to a tertiary care hospital to determine areas that need to be addressed. Furthermore, the instrument developed had a very strict cutoff values for both validity and reliability measures. Therefore, only a limited number of items could be included in the final checklist. Since the medical call writing trends of regional tertiary care hospitals was explored, we could not calculate the statistical generalizability.

## CONCLUSION

Medical call writing is an important but often neglected component of patient management. We first developed consensus of clinicians from different specialties on the minimum essential information in a medical call and then evaluated written calls from three teaching tertiary care hospitals. The study showed that most of our medical calls do not contain minimum essential information for patient management that may be attributed largely to the lack of formal training at under-and postgraduate level. Furthermore, a structured template including all the essential information needs to be implemented to ensure better call writing trend for patient management.

### Authors Contribution

**ARK:** conceived, designed and did data collection, statistical analysis and manuscript writing.

**UM:** conceived the idea, designed the instrument, helped in statistical analysis, did editing, reviewed all drafts and finally approved the manuscript.

**NB:** searched literature, interpreted results, revised manuscript, and reviewed and edited the final draft

All authors are responsible and accountable for the accuracy and integrity of the work.
